# Does timing of sexual debut following menarche among female youth in Uganda matter? A discrete time analysis

**DOI:** 10.1186/s12905-024-03201-0

**Published:** 2024-06-17

**Authors:** Dick Nsimbe, Charles Lwanga, Hellen Namawejje

**Affiliations:** 1 Department of Statistical Methods and Actuarial Science, School of Statistics and Planning, Makerere University, Kampala, Uganda; 2 Department of Population Studies, School of Statistics and Planning, Makerere University, Kampala, Uganda

**Keywords:** Sexual debut, Menarche, Uganda

## Abstract

**Background:**

The burden of early sexual engagement among youth is enormous. It directly raises the risk of sexually transmitted infections(STIs) and indirectly contributes to unintended pregnancy, unsafe abortion, premature childbirth, and psychosocial issues. The aim of this paper was to estimate the timing of sexual debut and examine the factors influencing the timing of first sexual intercourse following menarche among female youth aged between 15 and 24 in Uganda.

**Method:**

Self-reported data were extracted from the 2016 Uganda Demographic and Health Survey (UDHS), with a sub-sample of 7964 female youth from the individual woman file. Kaplan-Meier survival curves, decrement life-table analysis, and the discrete-time logit model were used to examine the timing of sexual debut and associated factors.

**Results:**

67.4% of the female youth had experienced first sexual initiation. Overall, the meantime to sexual debut was 4.4 years and the median time was 4.3 years, and all the female youth had experienced first sexual initiation by the end of the twelfth year following menarche. Significant factors found to influence the timing of sexual initiation include having higher education level (OR = 0.724: 95% CI = 0.59–0.89; *p* = 0.003), residing in the Northern region (OR = 0.877:95% CI = 0.79–0.97, *p* = 0.012), being employed (OR = 1.085: 95% CI = 1.01–1.16; *p* = 0.021), and being literate (OR = 1.155; 95% CI = 1.07–1.25; *p* < 0.001).

**Conclusions:**

These findings are expected to be central in the bid to delay first sexual intercourse. Also they shed light on some of the factors associated with the timing of sexual debut which may be addressed at community level for non-school going youth and in schools, as school based prevention sexual and reproductive health programs. The findings highlight the need for future studies to collect more data to explore further the linkage between time to first debut since menarche and, mass media, religion, type of residence, and wealth index.

**Supplementary Information:**

The online version contains supplementary material available at 10.1186/s12905-024-03201-0.

## Background

Sexual debut and its timing among youth continue to attract attention because it signals the beginning of exposure to a variety of sexual and reproductive health outcomes [1]. The environment under which the event occurs may have repercussions for the female youths’ future sexual behaviour and general health [2]. Initiation of first sexual intercourse is a key social developmental transition of youth, related to physical maturation, cognitive development, increasing awareness and appreciation of one’s body; consolidation of personal and sexual identity, and sexual relationship formation [3]. Individuals should have access to information and resources to make informed decisions about their sexual lives. Deciding to wait until one is fully ripped means taking control of one’s body and future, according to current national norms, a first sexual intercourse is early if it occurs before the age of 15, normative if it occurs between the ages of 15 and 19, and late if it occurs after the age of 19 [4]. Globally, United Nations defines youth as an individual in the age group of 15–24 years [5]. 16% of the world’s population are youth aged 15–24 years contributing to over 1.2 billion [6]. 11% of Asian female youth have had their first sexual intercourse by age 18, 44% of Latin American female youth by age 16, and 52% of sub-Saharan African female youths by age 19, and in developed countries, most female youths have had their first sex before age 20, which contributes to 67% in France, 79% in Great Britain, and 71% in the United States [7]. Furthermore, based on a 2011 UNICEF survey, in 10 out of 12 developed nations with data, over two-thirds of youth have had their first sexual intercourse while still in their teens [8]. In Denmark, Finland, Germany, Norway, Iceland, the United Kingdom and the United States, the proportion is approximately 80%. In Australia, the United Kingdom and the United States, approximately 25% of 15-year-olds and 50% of 17-year-olds have had their first sexual intercourse [8].

In Africa, youth have remained at a higher risk of early sexual outcomes, such as pregnancy and sexually transmitted diseases [9]. In Sub-Saharan Africa, the last decade has seen significant progress in decreasing risky sexual behaviors among youth [10]. Specifically, most youth’s first sexual intercourse now occurs between the ages of 15 and 24 [11]. In East Africa, the prevalence of early sexual initiation is approximately 58% [12]. The Demographic and Health Survey of Uganda, Kenya, and Tanzania, the study’s findings suggested that Ugandan women have sexual intercourse at the youngest age compared to Kenya and Tanzania. According to the survey, Uganda’s median age at sexual debut is 16.4 years, which is at least a year younger than Tanzania, which has a median age of 17.4 years, and nearly two years younger than Kenya, which has a median age of 18.2 years [13]. Therefore, there is a great need to model factors that are associated with the timing of first sexual intercourse as female youth progress from menarche. Understanding the factors influencing the age at which youth initiate sexual activity following menarche is crucial for implementing effective interventions and targeted educational programs. Early sexual debut can have significant implications for reproductive health, including increased risks of unintended pregnancies and sexually transmitted infections (STIs). By identifying and comprehending these factors, policymakers and healthcare providers can develop strategies to promote healthy behaviors and empower youth to make informed decisions about their sexual health.

In Uganda, youth are the youngest population in the world, with 77% of the population being under 25 years and more than 7.3 million youth aged 15–24 years [14]. According to the 2016 Uganda Demographic and Household Survey (UDHS), 10.3% of girls aged 15 to 19 in Uganda had initiated sexual activity by age 15. In the same research, the median age of sexual debut among female aged 20 to 49 is 17.1 years, with 18% having their first sexual contact before 15 years. 62% had their first sexual intercourse by 18 years, and 83% had their first sexual encounter by the age of 20 [15]. Furthermore, youth especially girls, are now at a greater risk of illness and death from reproductive causes, such as sexually transmitted infection, HIV, early pregnancy, and unsafe abortion [16]. Most studies have identified the level of education, wealth index status, religion, and mass media as the most prevalent factors influencing sexual debut [11, 17–20]. For Uganda to achieve Target 3.7 of the United Nations Sustainable Development Goals (SDGs), which urges countries to strive for universal access to sexual and reproductive health-care services by 2030, it must prioritize efforts to ensure universal access to sexual and reproductive health-care services, including family planning, education, and information [21, 22]. This study aligns with broader global objectives outlined in the SDGs, emphasizing the importance of addressing issues related to youth sexual reproductive health to achieve specific targets related to health, gender equality, and reducing inequalities. Additionally, integrating reproductive health into national strategies and programs is essential to empower individuals, particularly youth, to make informed decisions about their reproductive health in line with the broader SDG framework. It is against this background that this study hoped to investigate factors on the timing of first sexual intercourse following menarche among female youth aged 15–24 years in Uganda to inform the design of appropriate sexual and reproductive health programs and interventions.

## Theoretical consideration

A number of theories and research findings have attempted to explain youth behavior in relation to sexual debut. Example of such theories include the problem behavior theory, social learning theory, and the theory of planned action. These theories have been used by different scholars to explain sexual behavior among the adolescence [23, 24]. According to Problem Behavior Theory, some behavior is only problematic if it is defined as such by the culture in which the teenager is entrenched. As a result, the correlations between sexual initiation and other characteristics may vary if civilizations differ in their understanding of early sexual debut among youths as undesirable [25]. Alternatively, based on teenagers’ physical and mental immaturity, relatively early sexual initiation may be more uniformly hazardous regardless of the cultural setting [26]. According to social learning theory, some people on this spectrum have a completely internal or entirely external locus of control, but many will have some balance of both views, which may vary depending on the situation. People who have a high internal locus of control believe in their ability to control themselves and influence their surroundings. They believe that their future is in their own hands and that their choices determine whether they succeed or fail. One disadvantage of an internal locus of control is that accepting responsibility entails accepting blame for failures [27].

According to the theory of planned action, a person’s conduct is governed by the intention to carry out the activity, which results from the attitude toward the action and subjective norm. The intention is the most accurate predictor of behavior regarded to be the immediate antecedent of conduct, since it is the cognitive representation of a person’s readiness to engage in certain behavior. This purpose is influenced by three factors: their attitude toward specific activity, their subjective standards, and their perceived behavioral control [23]. A young person’s notion that another person’s view on sexuality is important in determining whether to partake in the behavior might be seen as a subjective norm. If the youth’s parents do not want her to have sex but the likelihood of them finding out is low, and friends will look up to her if she has sex, the motivation to comply with the friends is likely to be greater than the motivation to comply with the parents, resulting in a positive subjective norm towards having sex.

Building on the arguments behind these theories, this study is guided by the planned behavior action to investigate the factors influencing the timing of sexual debut from menarche among female youth aged 15–24 in Uganda. We tested three hypotheses: First, female youth in rural areas are more likely to have sexual debut earlier than those in urban areas. Second, female youth who are poor are more likely to have their first sex earlier than those who are from rich; and third, female youth from the Central region are likely to have first sex earlier than those from Eastern, western and Northern regions.

We hypothesized that the physiological changes occurring during puberty (rather than menarche itself) were associated with age of sexual debut in this population. Pubertal development typically starts at 8–13 years in girls, and the first stages of puberty usually precede menarche by approximately 2.5 years [28]. Early puberty may lead to early first sex because hormonal changes make girls interested in sexual activity at a younger age and because the associated bodily changes make them more attractive to males. In some local cultures, menarche itself is seen as a marker of womanhood, after which girls are expected to enter into relationships or marriage [29, 30]. However, no girls in this study were married and there was no effect of tribe or religion on the association between menarche and sexual debut. We considered it possible that pressure or force at first sex may mediate the relationship between early puberty and early sexual debut in this population. However, the association between age of menarche and early sexual debut observed in our study was even stronger after excluding girls reporting pressure or force at first sex (though under-reporting of coercion cannot be ruled out).

## Methods

With permission from the ICF International website, data were obtained from the 2016 Uganda Demographic and Health Survey (UDHS). The DHS surveys are currently part of the worldwide series of nationally representative cross-sectional household surveys. The original survey consisted of a selection of a representative sample of 20,880 households using a two stage sampling procedure. In the first stage, 697 enumeration areas (EAs) (162 from urban & 535 from rural) were selected from the 2014 Uganda national population and housing census (NPHC). The second stage comprised of listing households in each of the 697 accessible selected EAs. All women aged 15–49 found in the household the night before the survey were eligible to be interviewed; and out of the 19,088 eligible women, 10,117 women were interviewed. It is from these that a subsample of 7964 females aged 15–24 was extracted. The female youth were asked whether they had been initiated to first sexual intercourse or not. The age range of 15–24 years was chosen for three reasons: first, to reduce the effects of time; second, this age range is also very homogenous in terms of cohort analysis; and third, by the age of 24, most females may have had sex for the first time.

### Measures of outcome

The dependent variable in this study was the time to sexual debut after menarche. Respondents were asked, ‘*How old were you when you had your first sexual intercourse*?’. As a results, the dependent variable was created as the difference between the actual age of the female youth at first sex and the average age at menarche which is estimated to be 12 years [6]. All female aged 25 and above and had not had their first sexual intercourse were right censored. Right censoring happens when the event has not happened by the end of the observation period.

### Measures of explanatory variables

The independent variables were the socioeconomic and exposure characteristics of female youth. They include religious affiliation categorized as Catholic, Anglican, Muslim, and others; level of education recorded as no education, primary, secondary, and a higher level of education; region of residence categorized as: Central, Eastern, Northern and Western; type of place of residence recorded as urban and rural; occupation status was modelled as, not-working and Working; wealth Index of the household grouped as poor, middle, and rich. Media access grouped as having access to media and otherwise. Literacy status was thought to be distinct from the level of education because literacy primarily involves the acquisition of the ability to read and write, while education is concerned with the holistic development of a person. In modelling, this was categorized as can read and write, and cannot read and write.

### Statistical analysis

Lifetable estimates, Kaplan-Meier survival curves and the discrete-time logit model were used to estimate the timing of sexual debut following menarche and also identify the risk factors associated with the timing of sexual debut. The decrement lifetable was used to estimate the proportion of female youth transiting to first sexual intercourse following menarche [31]. Besides, lifetables were used to examine whether differences sexual initiation risks resulting from differences within covariates converge overtime. A generalized Wilcoxon test, which is a non-parametric statistical tool, set at *p* < 0.05, was used to test for differences between survival functions for different groups. The Kaplan-Meier (KM) survival curve was used at the bivariate level to estimate the median and mean time to first sexual debut and the significant difference in the mean and median time to sexual initiation arising from changes in covariates was assessed using the log-rank chi-square test with a 5%. Before fitting the discrete-time logit model, data was transformed from person oriented to period oriented. Data was transformed to track the time when the event of sexual debut would occur. The discrete time logit model was used to identify net effect of the risk factors associated with the timing of sexual debut among female youth, which is usually estimated as ratio of youth experiencing first sexual intercourse at the end of the interval to those who had attained menarche. The fitted model was subjected to the *linktest* for two reasons: First, to examine whether the independent variables were specified correctly; and second, to assess the goodness of fit of the model [32]. The *linktest* use the *hat* and _*hatsq statistic* and when the model describes the data well, the *_hatsq* should not be significant (*p > 0.*05), which implies that the observed data is similar to the expected. Before fitting the model, the independent variables were tested for collinearity (results not presented) and all the highly correlated variables were excluded from the model. In this study, missing data is assumed to be missing completely at random and therefore does not bias inferences [33].

## Results

Table 1 presents the prevalence, mean and median time, to sexual debut by socioeconomic and exposure characteristics. Overall, approximately 67% of the female youths in the study had their first sexual intercourse. A total of 5367 female youth aged 15–24 years were enrolled and of these, 29% were from the Eastern region and about 23% were from the Northern. The majority (76%) were from rural areas. Nearly 40%, 31%, 14% and 15% of the female youth were affiliated to the Catholic, Anglican, Muslim and other minority religious groups, respectively. Table 1 further shows that most of the respondents were of primary level education (61%) and about 3% had no formal education. Approximately 65% of respondents were working. With regard to wealth index, about 43% of the female youth were from poor households and nearly 17% were from the middle household status. The majority (73%) could read and write, and 79% were accessing information through radios, televisions, or newspapers.

### Mean and median time to sexual debut following menarche

**Fig. 1:**
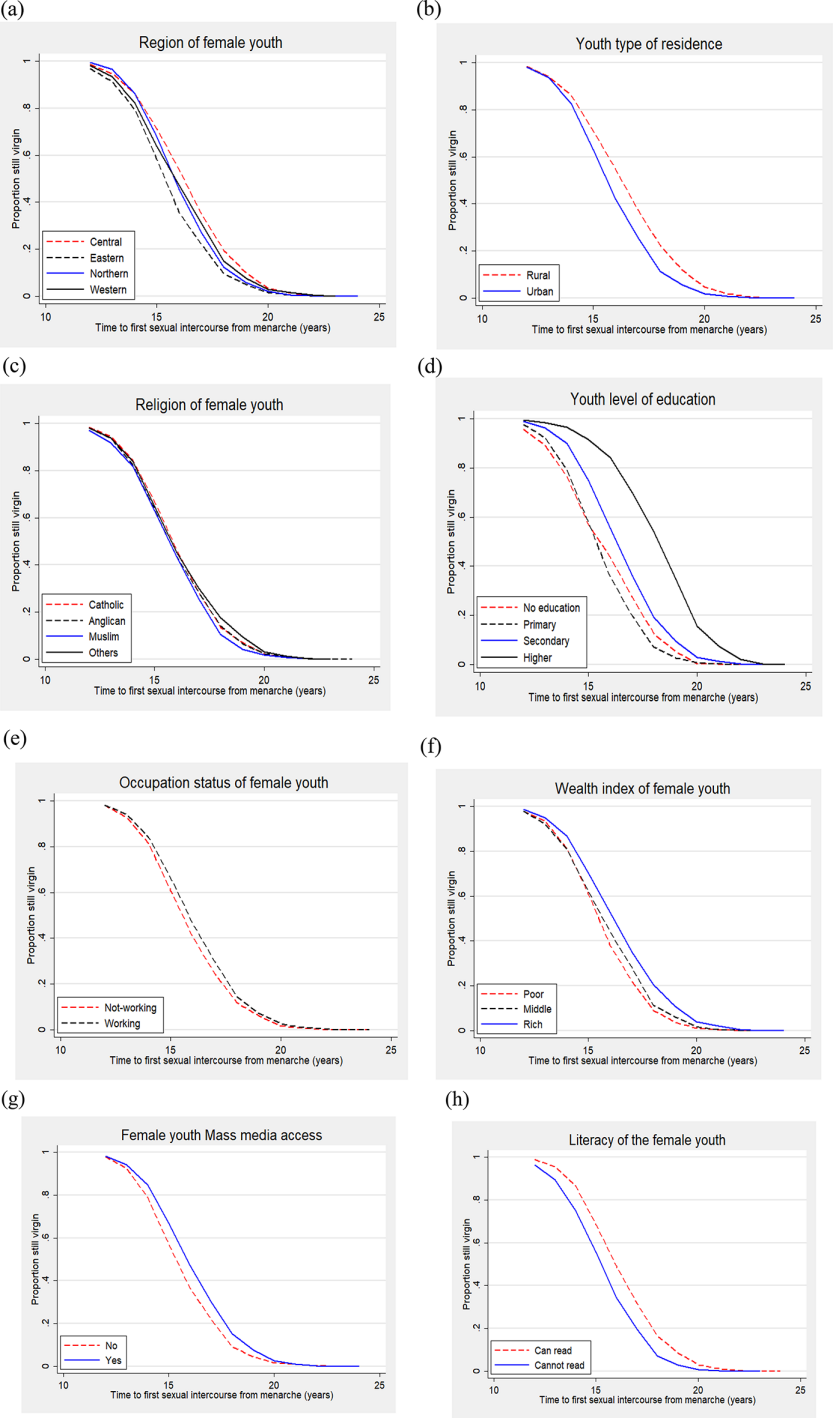
Kaplan-Meier plots showing the proportion of female youth who were still virgins by selected socio-demographic, economic, and exposure factors: (**a**) Region of residence, (**b**) type of residence, (**c**) religious affiliation, (**d**) Level of education, (**e**) occupation status, (**f**) wealth index, (**g**) Mass media, (**h**) Literacy status

Table 1; Fig. 1 present the Kaplan-Meier. Figure 1 shows that initiation of first sexual intercourse happened few years after menarche and increased over time. The overall mean and median time to first sexual intercourse was found to be about 4.4 and 4.3 years respectively. The meantime and median time to first sexual intercourse varied by region of residence, with female youth in the Central region, on average spending longer to have their first sexual intercourse than female youth either in the Eastern, Western and Northern regions. Female youths from urban areas had their first sexual intercourse at least a year later than those in the rural. Regarding religious affiliation, the Catholic female youths took longer to have their first sexual intercourse compared to Muslim or Anglican female youths.

**Table 1:** Differentials in the proportion of female youth having sexual debut and timing of sexual debut by selected socio-demographic, economic and exposure factors

Covariate	*N*	Percentage of first sexual intercourse from menarche	Mean time (years) to first intercourse (Kaplan Meier estimate)	Median time (years)to first intercourse (Kaplan Meier estimate)	Log-rank *chi*^2^(*χ*^2^), *p*
**Region of residence**					
Central	1,293	24.1	4.7	5.0	
Eastern	1,588	29.6	4.0	4.0	
Northern	1,219	22.7	4.4	4.0	96.7
Western	1,267	23.6	4.4	4.0	(*p* = 0.000)
**Type of Residence**					
Urban	1,246	23.6	4.8	5.0	91.2
Rural	4,099	76.4	4.2	4.0	(*p* = 0.000)
**Religion**					
Catholic	2,161	40.3	4.4	4.0	
Anglican	1,638	30.5	4.3	4.0	
Muslim	739	13.8	4.2	4.0	10.3
Others	829	15.5	4.5	4.0	(*p* = 0.015)
**Level of education**					
No education	204	3.8	4.1	4.0	
Primary	3,226	60.1	3.9	4.0	
Secondary	1,574	29.3	4.9	5.0	603.5
Higher	363	6.8	6.5	7.0	(*p* = 0.000)
**Occupation**					
Not working	1,341	25.0	4.2	4.0	16.3
Working	4,015	74.9	4.4	4.0	(*p* = 0.000)
**Wealth Index**					
Poor	2,313	43.1	4.1	4.0	
Middle	923	17.2	4.3	4.0	150.0
Rich	2,131	39.7	4.8	5.0	(*p* = 0.000)
**Mass Media**					
No	1,138	21.2	4.0	4.0	46.9
Yes	4,229	78.8	4.4	4.0	(*p* = 0.000)
**Literacy**					
Can Read	3,914	72.9	4.6	4.0	159.7
Cannot Read	1,453	27.0	3.8	4.0	(*p* = 0.000)
All	5,367	67.4	4.4	4.3	

Kaplan-Meier estimates; log rank *χ*^2^ test

Furthermore, Table 1 shows that female youth who had a secondary level of education or higher took at least a year longer before having their first sexual intercourse than those with primary or with no education. Not-working female youth had their sexual debut earlier than those who were working. Regarding the wealth index, female youth from poor households had their first sexual intercourse a year earlier than those from rich households. Regarding mass media, youth who had access to the media had their first sexual intercourse later than those who never had access. Female youth who could not read and write had their sexual debut a year earlier than those who could. This description can also be observed from the Kaplan-Meir curves as they spread outwards.

### Sexual debut since menarche

Table 2 presents lifetable estimates showing the proportion of female youth transiting from menarche to first sexual intercourse by some selected socioeconomic and exposure factors in Uganda. The relationship between time to first sexual intercourse and region of residence shows a significant difference in female youth for regions of residence (Wilcoxon-Gehan = 89.18, *p* = 0.0000). These analyses show that about only 19%, 9%, 12% and 15% of the female youth from the Central, Eastern, Northern, and Western regions had not gotten their sexual debut. Table 2 further shows an earlier transition to first sexual intercourse following menarche for females from rural areas compared to those in the urban (Wilcoxon-Gehan = 72.52, *p* = 0.0000). Table 2 shows no evidence of a significant difference religious affiliation with regard to sexual debut after menarche (Wilcoxon-Gehan = 5.63, *p* = 0.1283). In addition, Table 2 show that by the end of the seventh year following menarche, less than 20% had not gotten first sexual intercourse. These data also emphasize the view that, overall, education postpones intimate love making for females. The relationship between level of education and sexual debut showed a significant difference between female youth with less than secondary education and those with secondary education or higher. About 57% of females with no education had been initiated into first sexual intercourse by the end of the fifth-year following menarche compared to only 16% of female youth with a higher level of education (Wilcoxon-Gehan = 518.75, *P* = 0.0000). With respect to occupation status, non-working female youth significantly accelerated the transition from menarche to first sexual intercourse. By the end of the sixth year, 76% of female youth who were not working had been initiated into first sexual intercourse compared to 71% of females who were working (Wilcoxon-Gehan = 19.03, *p* = 0.0000). Table 2 also indicates that being poor significantly influenced sexual debut among female youth following menarche. Data shows that 62% of female youth from poor households had experienced first sexual intercourse compared to 48% of the females from rich households (Wilcoxon-Gehan = 114.16, *p* = 0.0000).

**Table 2:** Decrement life-table estimates showing the proportion of female youth transiting to first sexual intercourse following menarche by selected socio-demographic, economic, and exposure factors among 15-24-years female youth in Uganda

						Years since Menarche							
Covariates	1	2	3	4	5		6	7	8	9	10	11	12
Region*													
Central	0.99	0.95	0.86	0.71	0.54		0.35	0.19	0.10	0.03	0.01	0.00	0.00
Eastern	0.97	0.91	0.79	0.58	0.35		0.22	0.09	0.05	0.02	0.01	0.00	0.00
Northern	0.99	0.96	0.86	0.68	0.45		0.27	0.12	0.05	0.02	0.01	0.00	0.00
Western	0.98	0.93	0.82	0.64	0.47		0.31	0.15	0.07	0.03	0.02	0.01	0.00
Type of Residence*													
Urban	0.98	0.93	0.86	0.70	0.54		0.37	0.22	0.11	0.04	0.02	0.01	0.00
Rural	0.98	0.93	0.82	0.63	0.41		0.25	0.11	0.05	0.02	0.01	0.00	0.00
Religion***													
Catholic	0.98	0.94	0.84	0.66	0.47		0.29	0.14	0.07	0.02	0.01	0.00	0.00
Anglican	0.98	0.94	0.82	0.64	0.43		0.28	0.14	0.06	0.02	0.01	0.00	0.00
Muslim	0.97	0.91	0.81	0.62	0.43		0.25	0.10	0.04	0.02	0.00	0.00	0.00
Others	0.98	0.93	0.83	0.64	0.45		0.30	0.17	0.09	0.03	0.01	0.00	0.00
Level of education*													
No education	0.96	0.89	0.76	0.57	0.43		0.27	0.12	0.05	0.00	0.00	0.00	0.00
Primary	0.97	0.92	0.79	0.58	0.36		0.20	0.07	0.02	0.01	0.00	0.00	0.00
Secondary	0.98	0.96	0.90	0.75	0.56		0.36	0.19	0.09	0.03	0.01	0.00	0.00
Higher	0.99	0.98	0.96	0.91	0.84		0.69	0.53	0.34	0.15	0.07	0.02	0.00
Occupation*													
Not-working	0.97	0.92	0.81	0.60	0.40		0.24	0.11	0.06	0.01	0.00	0.00	0.00
Working	0.98	0.94	0.83	0.66	0.46		0.29	0.14	0.07	0.02	0.01	0.00	0.00
Wealth Index*													
Poor	0.97	0.93	0.81	0.61	0.38		0.22	0.09	0.03	0.01	0.00	0.00	0.00
Middle	0.97	0.92	0.80	0.61	0.44		0.28	0.11	0.06	0.01	0.00	0.00	0.00
Rich	0.98	0.94	0.86	0.70	0.52		0.35	0.20	0.10	0.04	0.02	0.00	0.00
Mass Media*													
No	0.97	0.92	0.78	0.57	0.36		0.22	0.09	0.04	0.01	0.01	0.00	0.00
Yes	0.98	0.94	0.84	0.67	0.47		0.30	0.15	0.07	0.02	0.01	0.00	0.00
Literacy*													
Can Read	0.98	0.95	0.86	0.68	0.48		0.31	0.16	0.08	0.03	0.01	0.00	0.00
Cannot read	0.96	0.89	0.74	0.55	0.34		0.19	0.07	0.02	0.00	0.00	0.00	0.00

Wilcoxon-Gehan; *p < 0.001; **p < 0.05; ***p < 0.10

Regarding access to mass media and being literate, the trend is that having access to information and being literate negatively influenced the timing of sexual debut. Table 2 shows that 64% of females with no access to mass media had by the end of the fifth year had had sexual debut compared to 53% of those with access to the media (Wilcoxon-Gehan = 54.65, *p* = 0.0000). Furthermore, 66% of the female youth who could not read and write had experienced first sexual intercourse compared to 52% of those who could read and write (Wilcoxon-Gehan = 131.16, *p* = 0.0000).

### Risk factors for time to sexual debut since menarche

In identifying the net effect of each independent factor on the time to sexual debut since menarche, a discrete-time logit model was built with the help of identified risk factors explained by the bivariate analysis as shown in Table 3. In this case, all significant independent factors at the bivariate level were included in the model. These include region of residence, type of residence, religious affiliation, level of education, literacy status, mass media, occupation status, and wealth index. Table 3 shows that the odds of experiencing sexual debut since menarche decreased for female youth from the Northern and Western region compared to the Central, but increased for females from the Eastern region. Again Table 3 shows that female youth from the Northern region were 12% less likely compared to the Central (OR = 0.877; 95% CI = 0.79–0.97, *p* = 0.012). With respect to the Western, females were 4% less likely but insignificant (OR = 0.962; 95% CI = 0.87–1.05, *p* = 0.407). The odds of experiencing sexual debut since menarche decreased among female youth with increase in education level; primary level (OR = 1.041; 95% CI = 0.88–1.23, *p* = 0.638); secondary level (OR = 0.901; 95% CI = 75-1.08, *p* = 0.267); and higher or above (OR = 0.724; 95% CI = 0.59–0.89, *p* = 0.003) compared with those with no education. Furthermore, there was an increased likelihood of initiating first sexual intercourse following menarche for female youth who could not read and write (OR = 1.155; 95% CI = 1.07–1.25, *p* < 0.001) compared to those who are literate. Female youth who were working were 9% more likely to have experienced first sexual intercourse since menarche compared to those who were not (OR = 1.085; 95% CI = 1.01–1.16, *p* = 0.021). Although there was no significant difference between female youth of different religious denominations, females affiliated to the Muslim faith (OR = 1.078; 95% CI = 0.98–1.19, *p* = 0.118); and those affiliated to Anglican Church (OR = 1.014; 95% CI = 0.94–1.09, *p* = 0.714) were found to be more likely to have sexual debut than those affiliated to the Catholic Church. Regarding the diagnostic test, the specification error results indicate that the discrete logit model was well specified (*hat: p = 0.09; _hatsq: p = 0.342*).

**Table 3:** Discrete time logit regression results linking the time to sexual debut to socioeconomic and exposure characteristics among female youth aged 15–24 in Uganda (2016)

Covariate	Odds Ratio (OR)	*P*-value	95% CI
**Region**			
Central (Ref.)	1.000	-	-
Eastern	1.054	0.262	0.96, 1.15
Northern	0.877	**0.012**	0.79, 0.97
Western	0.962	0.407	0.87, 1.05
**Type of Residence**			
Urban (Ref.)	1.000	-	-
Rural	1.000	0.999	0.92, 1.09
**Religion**			
Catholic (Ref.)	1.000	-	-
Anglican	1.014	0.714	0.94, 1.09
Muslim	1.078	0.118	0.98, 1.19
Others	0.971	0.524	0.89, 1.06
**Level of education**			
No education (Ref.)	1.000	-	-
Primary	1.041	0.638	0.88, 1.23
Secondary	0.901	0.267	0.75, 1.08
Higher	0.724	**0.003**	0.59, 0.89
**Occupation**			
Not working (Ref.)	1.000	-	-
Working	1.085	**0.021**	1.01, 1.16
**Wealth Index**			
Poor (Ref.)	1.000	-	-
Middle	0.959	0.356	0.88, 1.04
Rich	0.942	0.170	0.86, 1.03
**Mass Media**			
No (Ref.)	1.000	-	-
Yes	0.992	0.852	0.92, 1.07
**Literacy**			
Can read (Ref.)	1.000	-	-
Cannot Read	1.155	**0.000**	1.07, 1.25
-Log likelihood			14285.7
Number of subjects			5367
Total time at risk			31,406

(Ref.) = reference category, CI = confidence interval

## Discussion

Early initiation of sexual intercourse among adolescents exposes them to more sexual partners and a long period of sexual activity. This exposure contributes significantly to the social and public health problems. In many cases, adolescents consider first sexual experience as the point which signifies the transition to adulthood. Consequently, to the youth, sexual debut is approached with a combination of anticipation and anxiety. This study aimed to specifically estimate the timing of sexual debut and also examine the factors which influence sexual debut following menarche in Uganda. We tested three hypotheses: First, female youth in rural areas are more likely to have sexual debut earlier than those in urban areas; Second, female youth who are poor are more likely to have their first sex earlier than those who are rich; Third, female youth from the Central region are likely to have first sex earlier than those from other regions. Results from the study show that the mean time to first sexual intercourse is about 4.4 years and the median is 4.3. Result from the analysis further show that residing in the Northern and Western region, and having secondary education or higher are associated with reduced odds of having experienced first sexual intercourse and a delay in the timing of first sex. However, residing in the Eastern region, having primary education level, working, and being illiterate are associated with increased odds and an earlier timing of first sexual intercourse. Youth with secondary education or higher were found to be less likely to have their first sexual intercourse than those with no education. These results are consistent with those from other African countries. For instance, in the study conducted in Nairobi-Kenya, Karibu and Pamela found uneducated youth to be more likely to start sexual intercourse at an early age compared with their peers who are highly educated [34]. Similar results were found among Nigerian youths [20, 35]. This finding can be explained by three arguments: First, highly educated female youth can make educated decisions because they are informed about the impact of early sexual initiation [36]. Second, those who are educated have more freedom than those who are not. It is important to note that in Uganda, women with higher levels of education tend to delay marriage, which is a factor that contribute to delay of sexual debut. This is in contrast to females with little or no education who may marry at a younger age, and consequently, initiate sexual activity earlier [20]. Third, the agency of girls and their ability to make informed decisions are crucial components that contribute to education and delayed sexual debut. When girls are empowered to make choices about their reproductive health and future, they are more likely to prioritize education and delay their sexual debut [30]. This empowerment often comes through access to comprehensive sexuality education, information about reproductive health, and supportive environments that value girls’ autonomy and decision-making abilities [30, 37].

Our analysis reveals that female youth who were working are more likely to delay commencement of sexual activities compared to those not working. This is surprising because naturally, females irrespective of social status, can be lured by especially older male partners using little monetary gifts and subsequently get initiated into sexual activities [38] Thus, one would expect females that are working not to be enticed by such monetary gifts because they are economically better and as a result delay sexual debut than those that are not working. This finding is contrary to those of Rich and Kim who found an increased risk of sexual debut among unemployment youth [39], but in agreement with the findings of [35]. Two reasons may be used to explain these results: First, female youth who are working may be influenced by their workmates to engage in sexual intercourse in order to be liked or respected by their workmates more than those who are not working [40]. Second, as described earlier, regardless of a woman’s social status, exposure to earning money, may lead to the desire for more which force women to engage in sexual relationships for more money [20, 41].

An increase in the risk of experiencing first sexual initiation among females was found to be associated with illiteracy this is consistent with those of Beguy and colleagues who found out that youth with higher literacy levels are more likely to provide valid answers about the timing of first sexual intercourse than those with lower literacy levels [42]. Two perspectives may be used to explain these results: First, female youth who cannot read and write are unlikely to be aware of the risks of early sexual initiation [35]. Second, enables a person to develop basic cognitive skills, critical reflection, social awareness, and to apply these skills in lifelong learning, which helps a person to make informed reproductive decisions [43].

Regarding the region of residence of the female youth, results show that youth from the Northern and Western regions were found to be more likely to delay sexual debut compared to the Central region, however those from the Eastern region had an increased risk of experiencing first sexual initiation. These results are similar to those of [44] who investigated the relationship between urbanization, poverty and sexual behavior and found out that females in more urban areas are likely to experience early sexual initiation. Two arguments may be used to describe these results: First, in Uganda, the Central region is more urban than others. Urban area are commonly associated with slums whose residents demonstrate riskier sexual behavior and earlier sexual debut than non-slum residents [45]. Second, more urban areas are often affected by the effect of globalization and diffusion of ideas which alters conventional norms and causes changes in sociocultural characteristics such as religion, media exposure, education, and economic status. Thus these changes could explain why females’ sexual debut differ between different regions [46]. An increased risk for the Eastern region could be explained by high rates of child mothers and child marriages which might have increased the risk thus resulting in an earlier experience of sexual debut since it is almost impossible to postpone sexual initiation within marriage.

Wealth index of the household was used as a proxy for the parents’ economic status which often correlates with the youth’s sexual risky behaviour. While it was not found to be significant, implying that economic means of parents do not influence the timing of sexual debut, female youth living in middle and rich households would generally delay commencement of sexual activities. Religion, mass media, and type of residence which are known to influence sociocultural differences were in the present study found not to significantly influence the timing of sexual debut. However, with regard to the timing of sexual debut, female youth living in urban areas were as likely as their peers living in rural areas this could be due technological innovations which has made the flow of sex education information to reach rural areas the same way it does with Urban areas. Those who had access to mass media were 0.8% less likely to experience first sexual intercourse than their peers who lacked access.

In summary, the current study used the 2016 Uganda Demographic and Health Survey and survival analysis to investigate the timing of sexual debut following menarche among female youth aged 15–24. The study also examined associated factors using discrete time logit model and investigated the transition from menarche to first sexual initiation using the decrement lifetable. Accordingly, three hypotheses were tested, ‘Female youth in rural areas are more likely to have sexual debut earlier than those in urban areas’; ‘Female youth who are poor are more likely to have their first sex earlier than those who are from rich’; and ‘Female youth from the Central region are likely to have first sex earlier than those from other regions’. The discussion in the above seems to be in line with some perspectives behind the planned action theory the risk increased with female youth who were working and no significant influence for parent’s economic status. These results are expected to be central in the bid to delay first sexual intercourse. Relatedly, results are important as they shed light on some of the factors associated with the timing of sexual debut which may be addressed at community level for non-school going children and in schools, as school based prevention sexual and reproductive health programs. Also highlighted is that future studies should collect more data to explore further the linkage between time to first debut since menarche and, mass media, religion, type of residence, and wealth index.

There are some limitations which can be addressed in future studies. First, Sexuality is a complex construct, and age at first sexual intercourse was assessed using self-reported data, which may be subject to social desirability bias as some female youth could have wanted to look as if they were submissive to social norms. Second, the Uganda Demographic Healthy Survey data is cross-sectional. Thus, reported are factors associated with, which do not necessarily imply a causative relationship. In conclusion, the study found the mean time to sexual debut since menarche to be 4.4 years and median time was 4.3 years and all female youth had experienced first sexual intercourse by the end of the 12 years since menarche. Region of residence, level of education, occupation, and literacy were found to influence the timing of sexual debut following menarche.

### Electronic supplementary material

Below is the link to the electronic supplementary material.

Supplementary Material 1

## Data Availability

The data set used is openly available upon permission from MEASURE DHS website (URL: https://www.dhsprogram.com/data/available-datasets.cfm*).*

## Abbreviations

AFS: Age at First Sex
AIDS: Acquired Immunodeficiency Syndrome
CI: Confidence Interval
DHS: Demographic and Health Survey
EA: Enumeration Area
EAs: Enumeration Areas
HIV: Human Immunodeficiency Virus
KM: Kaplan-Meier
OR: Odds Ratio
PBT: Problem Behavior Theory
p-value/p: Probability Value
Std.Err: Standard Error
STIs: Sexually Transmitted Infections
UBOS: Uganda Bureau of Statistics
UDHS: Uganda Demographic and Health Survey
UN: United Nations
UNESCO: United Nations Educational, Scientific and Cultural Organization
UNFPA: United Nations Population Fund
UNHS: Uganda National Household Survey
UNICEF: United Nations Children’s Fund
USA: United States of America
USAID: United States Agency for International Development
WHO: World Health Organization

## References

[1] Yode M, LeGrand T (2012). Association between age at first sexual relation and some indicators of sexual behaviour among adolescents. Afr J Reprod Health.

[2] Ghebremichael M, Larsen U, Paintsil E (2009). Association of age at first sex with HIV-1, HSV-2 and other sexual transmitted infections among women in northern Tanzania. Sex Transm Dis.

[3] Santelli J et al. Prevalence of Sexual Experience and Initiation of Sexual Intercourse among Adolescents, Rakai District, Uganda, 1994–2011. https://www.ncbi.nlm.nih.gov/pmc/articles/PMC4671201/1/21. 2016. 10.1016/j.jadohealth.2015.07.018 PMC4671201 26499857

[4] Adimora DE, Onwu AO (2019). Socio-demographic factors of early sexual debut and depression among adolescents. Afr Health Sci.

[5] UNESCO. Welcome to the UNESCO Youth Programme. 2018.

[6] UNICEF. Menstruating girls in Adjumani can now finish school as UNICEF community barazas yield fruit. 2020. https://www.unicef.org/uganda/stories/menstruating-girls-adjumani-can-now-finish-school-unicef-community-barazas-yield-fruit-:~:text=Several%20researchers%20have%20concluded%20that,and%20child%20marriages%20in%20Uganda.

[7] Salgado A, Cheetham N. The Sexual and Reproductive Health of Youth: A Global Snapshot. 2003 https://www.advocatesforyouth.org/wp-content/uploads/storage/advfy/documents/fsglobal.pdf.

[8] UNICEF (2011). The effects of childhood sexual messages on African-American and white women’s adolescent sexual practice. Psychol Women Q.

[9] Onsomu E (2013). Delaying sexual debut as a strategy for reducing HIV epidemic in Kenya. Afr J Reprod Health.

[10] Woog V, Kågesten A. The sexual and reproductive health needs of very young adolescents aged 10–14 in developing countries: what does the evidence show. Guttmacher Institute. https://amaze.org/wp-content/uploads/2019/02/Resources_Gutmacher_20190219.pdf. 2017.

[11] Amo-Adjei J, Tuoyire D (2018). Timing of sexual debut among unmarried youths aged 15–24 years in sub- Saharan Africa. J Biosoc Sci.

[12] Ferede TA (2023). Prevalence and associated factors of early sexual initiation among youth female in sub-saharan Africa: a multilevel analysis of recent demographic and health surveys. BMC Womens Health.

[13] Njenga F. The East African. At 16, Ugandan girls lead in earliest age of first sexual experience. 2011. https://www.theeastafrican.co.ke/tea/news/east-africa/at-16-ugandan-girls-lead-in-earliest-age-of-first-sexual-experience-1304854-:~:text=Ugandan%20girls%20begin%20to%20have,than%20in%20Kenya%2C%20at%2018.2. 2011.

[14] Population, Action. The effect of a very young age structure in Uganda. 2010.

[15] Uganda Bureau of Statistics(UBOS). and ICF, Uganda Demographic and Health Survey 2016.Kampala, Uganda and Rockville, Maryland, USA. 2018.

[16] Kassahun EA (2019). Factors associated with early sexual initiation among preparatory and high school youths in Woldia town, northeast Ethiopia: a cross-sectional study. BMC Public Health.

[17] Mwangi LW. Factors influencing early sexual debut among 15–24 year old female youth in coast province, Kenya (Doctoral dissertation, University of Nairobi). 2014.

[18] Beguy D, Ndugwa R, Kabiru CW (2013). Entry into motherhood among adolescent girls in two informal settlements in Nairobi, Kenya. J Biosoc Sci.

[19] Marston M et al. Predictors of sexual debut among young adolescents in Nairobi’s informal settlements. Europe PMC Funders Group, 39. 2014. 10.1363/3902213 PMC4101799 23584465

[20] Fagbamigbe AF, Idemudia E (2017). Diversities in timing of sexual debut among Nigerian youths aged 15–24 years: parametric and non-parametric survival analysis approach. Afr Health Sci.

[21] Daher-Nashif S, Bawadi H (2020). Women’s health and well-being in the united nations sustainable development goals: a narrative review of achievements and gaps in the gulf states. Int J Environ Res Public Health.

[22] Biswas RK (2023). Contraceptive use in South and South-East Asian region: Assessment of sustainable development goal 3.7 through indicator 3.7. 1. J Public Health.

[23] Ajzen I (1991). The theory of planned behavior. Organ Behav Hum Decis Process.

[24] Rotter J. Social learning and clinical psychology. 1954.

[25] Awusabo A. Youth Sexual and Reproductive Health in Ghana: Results from the 2004 National Survey of Youths, Occasional Report. New York: Guttmacher Institute, 2006, No. 22. 2006.

[26] Jessor R (1987). Problem-behavior theory, psychosocial development, and youth problem drinking. Br J Addict.

[27] Rotter B. Internal Versus External Control of Reinforcement: A Case History of a variable. American Psychologist, April 1990, 490–493. 1990.

[28] Emmanuel M. B.B., Tanner stages. StatPearls. Treasure Island (FL): StatPearls Publishing LLC; 2020. 2020. 29262142

[29] Sunder N. Marriage age, social status, and intergenerational effects in Uganda. Demography. 2019; 56(6): 2123–46. 2019. 10.1007/s13524-019-00829-8 31792876

[30] Nash K (2019). Our girls need to see a path to the future--perspectives on sexual and reproductive health information among adolescent girls, guardians, and initiation counselors in Mulanje district. Malawi Reproductive Health.

[31] Preston S, Heuveline P, Guillot M (2001). Measuring and modeling Population processes.

[32] Kohler U, Kreuter F. Data Analysis Using Stata (Third Edition). Stata Press. 2012.

[33] Abonazel MR (2018). On estimation methods for binary logistic regression model with missing values. Int J Math Comput Sci.

[34] Karibu W, Pamela P. Factors associated with sexual activity among high-school students in Nairobi, Kenya. J Adolesc 1–17. 2008. 10.1016/j.adolescence.2008.08.001 18851878

[35] Hailegebreal S (2022). Prevalence and associated factors of early sexual initiation among female youth in East Africa: further analysis of recent demographic and health survey. BMC Womens Health.

[36] Cyan D, Rebecca L. Entertainment Television as a Healthy Sex Educator: The Impact of Condom-Efficacy Information, Paediatrics, Vol. 112, No. 5. 2013. 10.1542/peds.112.5.1115 14595055

[37] Geary CW (2013). Sexual agency and ambivalence in the narratives of first time sexual experiences of adolescent girls in Jamaica: implications for sex education. Sex Educ.

[38] Wamoyi J (2010). Transactional sex amongst young people in rural northern Tanzania: an ethnography of young women’s motivations and negotiation. Reproductive Health.

[39] Rich LM, Kim SB. Employment and the sexual and reproductive behavior of female adolescents. Perspect Sex Reprod Health, 127–34. 2002. 12137126

[40] Widman L (2016). Adolescent susceptibility to peer influence in sexual situations. J Adolesc Health.

[41] Guttmacher, Institute. Adolescents in Uganda: Sexual and Reproductive Health (Research In Brief Serie.2). https://www.guttmacher.org/sites/default/files/report_pdf/rib2-05.pdf.

[42] Beguy D (2009). Inconsistencies in self-reporting of sexual activity among young people in Nairobi, Kenya. J Adolesc Health.

[43] Global Monitoring EFA, UNESCO (2006). Report 2006. Education for all – literacy for life.

[44] Greif MJ, Dodoo FN-A, Jayaraman A (2011). Urbanisation, poverty and sexual Behaviour: the Tale of five African cities. Urban Stud.

[45] Mugisha F (2006). School enrollment among urban non-slum, slum and rural children in Kenya: is the urban advantage eroding?. Int J Educational Dev.

[46] Seff I, Steiner JJ, Stark L (2021). Early sexual debut: a multi-country, sex-stratified analysis in sub-saharan Africa. Glob Public Health.

