# Supplementary material for: Does timing of sexual debut following menarche among female youth in Uganda matter? A discrete time analysis
Source: BMC Womens Health. 2024 Jun 17;24:347. doi: 10.1186/s12905-024-03201-0 (PMC11181548; doi:10.1186/s12905-024-03201-0)
Supplement: Supplementary file 1 — Supplementary Material 1 [file 12905_2024_3201_MOESM1_ESM.docx]

Appendix One: Actual Age of first sexual intercourse
